# Transcriptome Analysis of the *Cf-12*-Mediated Resistance Response to *Cladosporium fulvum* in Tomato

**DOI:** 10.3389/fpls.2016.02012

**Published:** 2017-01-05

**Authors:** Dong-Qi Xue, Xiu-Ling Chen, Hong Zhang, Xin-Feng Chai, Jing-Bin Jiang, Xiang-Yang Xu, Jing-Fu Li

**Affiliations:** ^1^College of Horticulture, Northeast Agricultural UniversityHarbin, China; ^2^College of Life Science, Northeast Agricultural UniversityHarbin, China

**Keywords:** *Cladosporium fulvum*, resistance response, RNA-Seq, differentially expressed genes, *Cf-12* tomato

## Abstract

*Cf-12* is an effective gene for resisting tomato leaf mold disease caused by *Cladosporium fulvum* (*C. fulvum*). Unlike many other *Cf* genes such as *Cf-2, Cf-4, Cf-5*, and *Cf-9*, no physiological races of *C. fulvum* that are virulent to *Cf-12* carrying plant lines have been identified. In order to better understand the molecular mechanism of *Cf-12* gene resistance response, RNA-Seq was used to analyze the transcriptome changes at three different stages of *C. fulvum* infection (0, 4, and 8 days post infection [dpi]). A total of 9100 differentially expressed genes (DEGs) between 4 and 0 dpi, 8643 DEGs between 8 and 0 dpi and 2547 DEGs between 8 and 4 dpi were identified. In addition, we found that 736 DEGs shared among the above three groups, suggesting the presence of a common core of DEGs in response to *C. fulvum* infection. These DEGs were significantly enriched in defense-signaling pathways such as the calcium dependent protein kinases pathway and the jasmonic acid signaling pathway. Additionally, we found that many transcription factor genes were among the DEGs, indicating that transcription factors play an important role in *C. fulvum* defense response. Our study provides new insight on the molecular mechanism of *Cf* resistance to *C. fulvum*, especially the unique features of *Cf-12* in responding to *C. fulvum* infection.

## Introduction

*Cladosporium fulvum* (*C. fulvum*, syn. *Passalora fulva*) is a biotrophic pathogen of tomato (*Solanum lycopersicum*), which causes leaf mold disease (Cooke, 1883). This fungus infects primarily the foliage, and occasionally the petioles and stems (Butler and Jones, 1949; Jones et al., 1997). The infection often results in wilting leaves and defoliation, which reduce fruit yield and quality, and sometimes death of the entire plant (Thomma et al., 2005). *C. fulvum* has many physiological races, and new physiological races continue to be evolved (Westerink et al., 2004). These physiological races differ in race-specific elicitor proteins encoded by effector genes, and one of which is recognized as the AVR gene. The effector proteins are secreted into the apoplastic space during infection (Nekrasov et al., 2006), and induce either a compatible or incompatible interaction between the fungus and infected plant. An incompatible interaction (chlorosis) occurs when the plant is able to resist the pathogen and prevent infection, while a compatible interaction (necrosis) occurs when the pathogen is able to grow and ramify, causing necrosis to the infected cells (Hammond-Kosack and Jones, 1996). One of the most efficient containments is breeding *C. fulvum*-resistant tomato cultivars by introducing *Cf* resistance genes identified from wild *Solanum* species into cultivated tomato. More than 20 *Cf* genes have been identified since the discovery of the *Cf-1* gene in the 1930s (Lanford, 1937; Kanwar et al., 1980a), and these have been introduced into cultivated tomato (Kerr and Bailey, 1964; Kanwar et al., 1980a,b; Stevens and Rick, 1986; Dickinson et al., 1993; Jones et al., 1993; Joosten and de Wit, 1999; Haanstra et al., 2000; Zhao et al., 2016).

The recognition of plant to pathogen and its subsequent response is a complex and dynamic process (Joosten and de Wit, 1999; Rivas and Thomas, 2005). At least three layers of pathogen recognition/response mechanisms are present in plants. The first one is basal resistance, also known as innate immunity, which can be triggered by microbe-associated molecular patterns such as cell wall components found in microbes. When a pathogen suppresses the basal defense, plants may respond with a hypersensitive response (HR) characterized by deliberate cell death at the site of infection. The third layer of defense is called RNA silencing, where plants recognize and digest the DNA or RNA produced by viruses, making these unusable. *C. fulvum* penetrates the abaxial side of the leaf and secretes toxic avirulence proteins (*Avrs*) and extracellular proteins. These specific elicitors are recognized by *Cf* genes (Lauge et al., 1998) and trigger a hypersensitive response. As a result, these infected parenchyma and epidermal cells collapse and eventually form typical necrotic spots (Steinkamp et al., 1979; Feindt et al., 1981). Studies have unraveled the distinct recognition mechanism between *Cf* genes and *Avrs* such as the interaction between Cf-2 and Avr2 (Kruger et al., 2002; Luderer et al., 2002) and between Cf-9 and Avr9 (Koomangersmann et al., 1996). Studies have also revealed that gene expression patterns between *Cf-4/Avr4*- and *Cf-9/Avr9*-dependent defense responses have similar gene expression patterns (Romeis et al., 2001; Gabriëls et al., 2006; Nekrasov et al., 2006; Hong et al., 2007; van den Burg et al., 2008).

The *Cf-12* gene was identified through the large screening of *C. fulvum* resistant genes (Kanwar et al., 1980b). It is located at position 31 cM on chromosome 8 of the *Lycopersicon esculentum*. *Cf-12* is efficiently resistant to *C. fulvum*, but little is known about the molecular mechanism of its defense response. In addition, none of the physiological races of *C. fulvum* are virulent to *Cf-12* carrying plant lines, while many physiological races have been found to be virulent to other *Cf* genes, including *Cf-2, Cf-4, Cf-5*, and *Cf-9*.

RNA-Seq has been widely and successfully applied in biological analysis, particularly in plants (Hong et al., 2007; Varshney et al., 2009; Haas and Zody, 2010) such as wheat (Yang et al., 2015), rice (Bai et al., 2015), maize (Li et al., 2010), cabbage (Wang et al., 2016a), and cucumber (Zhang et al., 2014). In this study, the comprehensive transcriptome analysis of *Cf-12-*tomato at different infection stages was performed to identify differentially expressed genes (DEGs). The identified DEGs were further verified by qRT-PCR and analyzed using gene ontology (GO) and Kyoto Encyclopedia of Genes and Genomes (KEGG). Our results can help identify the key genes and pathways associated with *Cf-12*-mediated resistance response, and better understand the molecular mechanism of *Cf* resistance to fungal infection.

## Materials and methods

### Plants, *C. fulvum* strains, and pathogen infection

The resistant tomato line of *Cf-12-*tomato (CGN7495) and the susceptible line Moneymaker were obtained from the Chinese Academy of Agricultural Sciences (Beijing, China). They were grown in a greenhouse at the Horticultural station of Northeast Agricultural University (Harbin, China). The growing condition was 16-h light and 8-h darkness at 25°C with an ambient humidity of 95%. *C. fulvum* physiological race 1.2.3 was acquired from tomato-growing regions in Harbin using the single sporangiophore transfer method, as previously described (Hubbeling, 1971; Wong and Wilcox, 2000); and was further axenically propagated on Moneymaker. At the four-five leaf stage, the abaxial surface of the *Cf-12-*tomato seedlings was inoculated with a suspension of 1 × 10^7^ sporangia per ml (Wang et al., 2007). Leaf samples were harvested at 0–15 days of post-infection (dpi) for microscopic analysis.

### Microscopic observation of *C. fulvum* in *Cf-12* tomato

In order to observe the interaction process of *C. fulvum* on *Cf-12* tomato, the lactophenol trypan blue staining method was carried out according Franco's approach (Franco et al., 2008). The fungal tissue and dead host cells would be densely stained, while living host cells would impart a translucent and slightly brown color. The leaf samples were harvested at 0–15 dpi, immediately stained, clarified overnight in chloral hydrate solution (2.5 mg/ml) (Keogh et al., 1980), and examined using an EVOS® microscope (ThermoFisher, USA) and an OLYMPUS SZX10 dissecting microscope (Olympus, Japan).

### RNA extraction, library preparation, and sequencing

Total RNA from *Cf-12-*tomato leaves was extracted and analyzed, as previously described (Fang et al., 2015). The integrity of the isolated RNA was calculated, as previously described (Schroeder et al., 2006); and samples with an RNA integrity number >9.0 were used for libraries construction. The libraries were generated using the NEBNext® Ultra™ RNA Library Prep Kit for Illumina® (NEB, USA). The clustering of the index-coded samples was performed on a cBot Cluster Generation System using the TruSeq PE Cluster Kit v3-cBot-HS (Illumia). After cluster generation, the libraries were sequenced on an Illumina Hiseq 4000 platform conducted by the Novogene Bioinformatics Institute (Beijing, China); and 150-bp paired-end reads were generated.

### Quality control, mapping, and *de novo* assembly

Raw sequence data were processed using Perl scripts (http://www.perl.org), developed by Novogene Bioinformatics Institute (Beijing, China), in order to remove reads that contained adapter fragments and ploy-N stretches (the number of ploy-Ns is >10%). Phred quality scores (Q20: ratio of an error rate 1%, Q30: ratio of an error rate 0.1%) and GC-content were calculated, and only the data with a quality score (Q_phred_) ≥ 30 (Q30) were used for further analyses.

The reference genome and gene model annotation files were downloaded from the Ensembl Genomes Databases (ftp://ftp.ensemblgenomes.org/pub/release-23/plants/fasta/solanum_lycopersicum/dna/). An index of the reference genome was built using Bowtie v2.2.3 (Langmead and Salzberg, 2012), and paired-end clean reads were aligned to the reference genome using TopHat2 v2.0.12 (Kim et al., 2013). Cufflinks v2.1.1 (Trapnell et al., 2010, 2012) was used to construct and identify both known and novel transcripts from TopHat2 alignment results.

### Quantification and differential expression analysis of transcripts

HTSeq v0.6.1 (EMBL, Heidelberg, Germany) was used to count the read numbers mapped to each gene. Gene expression levels were calculated based on the length of the gene, sequencing depth and read count mapped to this gene using the Fragments Per Kilobase of transcript sequence per Millions base pairs sequenced (FPKM; Trapnell et al., 2010) method. Genes with FPKMs in intervals of 1–3, 3–15, 15–60, and beyond 60 were considered to be expressed at low level, medium level, high level and very high level, respectively.

After calculating the gene expression levels, a differential expression analysis of two conditions or groups was performed using the DESeq R package (1.18.0) (Anders and Huber, 2010). DESeq provides statistical strategies to determine differential gene expression using the negative binomial distribution model (K_ij_ ~NB[μ_ij_, $\sigma_{\text{ij}}^{2}$]). The resulting *P*-values and fold-changes were adjusted using the Benjamini-Hochberg's approach to control the false discovery rate (FDR ≤ DRe5; Benjamini and Hochberg, 1995). Genes with an adjusted *P*-value (*padj*) < 0.05 were considered to be significant DEGs.

### Validation of DEGs by quantitative real-time PCR

Eighteen DEGs involved in plant disease resistance pathways were validated using quantitative real-time PCR (qRT-PCR). The primer pairs of the selected genes were designed using Primer Premier 6.0 (Premier Biosoft, Canada; Table S1). The tomato *actin* gene (U60478.1) was used as a reference control. The qRT-PCR was performed using AceQ® qPCR SYBR® Green Master Mix (Vazyme, USA) on an iQ™ 5 Multicolor Real-time PCR Detection System (Bio-Rad, USA). The reaction parameters were as follows: 95°C for 7 min, and 40 cycles of 95°C for 10 s, 58°C for 30 s, and 72°C for 20 s. Each sample was repeated three times, and relative expression levels were evaluated using the 2^−ΔΔCt^ method (Livak and Schmittgen, 2001).

### GO and KEGG enrichment analysis of DEGs

The GO enrichment analysis of DEGs was performed using the GO-seq based on Wallenius non-central hyper-geometric distribution (Young et al., 2010), in which gene length bias in DEGs were adjusted by Bonferroni correction. GO terms with a corrected *P* < 0.05 were considered significantly enriched by DEGs. KEGG pathway enrichment (http://www.genome.jp/kegg/) was used to identify significantly enriched signal transduction pathways or metabolic pathways in DEGs. Significantly enriched DEG pathways were identified using the KOBAS 2.0 software (KOBAS, Surrey, UK; Xie et al., 2011), and adjusted by hyper-geometric test and Benjamini-Hochberg FDR correction (FDR ≤ F.05).

## Results

### Microscopy observation of *C. fulvum* invasion into *Cf-12* tomato leaves

**Figure 1:**
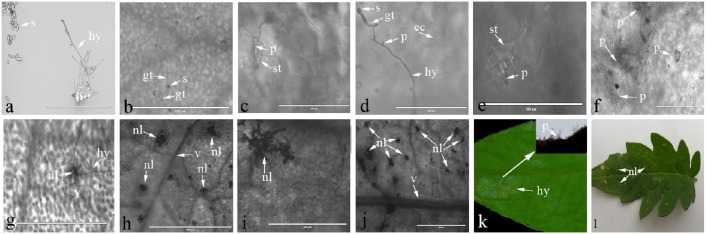
**Lactophenol trypan blue stained-tomato leaf samples after inoculation with ***C. fulvum***. (A)** Morphology of *Cladosporium fulvum* mycelium and spores; **(B)** conidiospores germinates (2 or 3 dpi); **(C,D)** the hypha penetrates into the stomata of the Moneymaker **(C)** and *Cf-2* tomato **(D)** (4 dpi); **(E)** the hyphae emerges through the stomata of the Moneymaker cultivar (8 dpi); **(F)** the hyphae increases and plugs the stomata on the Moneymaker cultivar (10 dpi); **(G)** cells surrounding the stoma had necrosis lesions on *Cf-12* tomato (8 dpi); **(H–J)** a large number of necrotic spots appeared and the necrosis area increased on *Cf-12* tomato (10–15 dpi); **(K)** thick gray-white mold layer on the abaxial surface of the infected leaf on the Moneymaker cultivar (15 dpi); **(L)** yellow necrotic spots on *Cf-12* tomato. s, spore; gt, germ tube; hy, hypha; p, penetration of the stomata by the hypha; ec, plant epidermal cell walls; v, host vascular tissue; st, stomata; nl, necrotic lesion.

Light microscopy was used to observe the interaction process between *C. fulvum* and *Cf-12* tomato, or the Moneymaker leaves. A representative image of the *C. fulvum* mycelium and spores are shown in Figure 1A. Our results revealed that conidiospores germinated at 2 or 3 dpi (Figure 1B), the hypha started penetrating into the stomata at 4 dpi in both the Moneymaker and *Cf-2* tomato (Figures 1C,D). No difference was observed between the two cultivars at this stage. Then, the hypha penetrated into the intercellular space and continued from the substomatal cavity into the intercellular space between the spongy mesophyll cells (Thomma et al., 2005), emerging through the stomata at 8 dpi and gradually plugging the stomata at 10 dpi on the Moneymaker cultivar (Figures 1E,F). However, cells surrounding the stomata appeared to die (necrosis) at 8 dpi on *Cf-12* tomato (Figure 1G); and a large number of necrotic spots began to appear at 10 dpi (Figure 1H). The area of necrosis was gradually enlarged at 12–15 dpi (Figures 1I,J). In the advanced stages of disease development, the hyphae formed a thick, gray-white mold layer on the abaxial surface of the leaves at 15 dpi on the Moneymaker cultivar (Figure 1K) and formed yellow necrotic spots on the front side of the leaf on *Cf-12* tomato (Figure 1L). This observation is in agreement with the results of a previous study (Thomma et al., 2005). Based on this observation, we selected *Cf-12* tomato samples at 0, 4, and 8 dpi for mRNA-Seq and qRT-PCR analysis.

### RNA sequencing and transcripts identification

In order to investigate differences in transcriptome between *C. fulvum-*infected *Cf-12* tomatoes (Cf12_B, 4 dpi; Cf12_C, 8 dpi) and non-infected controls (Cf12_A, 0 dpi), RNA from these three samples were sequenced. An average of 59,291,565, 56,131,442 and 70,822,873 raw reads from Cf12_A (0 dpi), Cf12_B (4 dpi), and Cf12_C (8 dpi) were generated, respectively (Table 1, Figure S1). After removing adaptors, low quality reads, duplications and ambiguous reads, an average of 60 million clean reads were obtained for each sample; and 94% of these clean reads were at the Q30 (ratio of error rate ≤ 0.1%) level (Table 1). Furthermore, at least 89% of these clean reads were mapped; of which, ~88% were uniquely mapped and 0.7% were multiple mapped to tomato chromosomes, respectively (Table S2). Pearson correlation coefficient (R^2^) analysis of the FPKM distribution between different biological replicates revealed a high level of reproducibility of RNA expression patterns (*R*^2^ = 0.98–0.99, *P* < 0.001; Table 2, Figure S2). A large majority of reads were mapped to the genome exon regions (Figure S3), and reads density was positively correlated to chromosome length (Figure S4). Cufflinks analysis revealed a total of 15,395 transcripts from the three samples (Cf12_A, Cf12_B, and Cf12_C), including 14,494 known transcripts (Table S3) and 901 new transcripts (Table S4).

**Table 1:** **Quantitative analysis of raw RNA-seq data**.

**Sample name**	**Raw reads**	**Clean reads**	**Clean bases (Gigabytes)**	**Error rate (%)**	**Q20 (%)**	**Q30 (%)**	**GC content (%)**
Cf12_A1	61,268,982	59,705,188	7.46	0.01	97.75	94.94	42.49
Cf12_A2	62,723,930	61,114,280	7.64	0.01	98.19	95.89	42.69
Cf12_A3	53,881,786	52,201,090	6.53	0.01	98.38	96.26	42.44
Cf12_B1	57,197,802	55,752,170	6.97	0.01	98.17	95.84	42.66
Cf12_B2	63,063,966	61,400,394	7.68	0.01	98.08	95.68	42.51
Cf12_B3	48,132,558	47,308,268	5.91	0.01	98.04	95.72	42.67
Cf12_C1	58,287,568	56,764,192	7.1	0.01	98.28	96.16	42.91
Cf12_C2	77,177,398	75,155,218	9.39	0.01	98.33	96.26	42.61
Cf12_C3	77,003,654	75,050,742	9.38	0.01	98.32	96.21	42.72

*Raw reads: the raw reads after transformation from the sequenced data by base calling. Clean reads: the reads of filtered raw reads. Clean bases: the clean bases after transformation from the sequenced data by base calling. Error rate: sequencing error rate. Q20: the bases amount ratio of error rate ≤ 1%. Q30: the bases amount ratio of error rate ≤ 0.1%. GC content: the amount of G & C in the total bases amount, %. A1–A3, B1–B3, and C1–C3 refer to three different samples from 0 (Cf12_A), 4 (Cf12_B), and 8 (Cf12_C) dpi, respectively*.

**Table 2:** **FPKM analysis of gene expression levels**.

**FPKM interval**	**Cf12_A1**	**Cf12_A2**	**Cf12_A3**	**Cf12_B1**	**Cf12_B2**	**Cf12_B3**	**Cf12_C1**	**Cf12_C2**	**Cf12_C3**
0~1	20,103 (50.72%)	20,231 (51.04%)	20,201 (50.97%)	20,626 (52.04%)	20,626 (52.04%)	20,239 (51.06%)	20,532 (51.80%)	20,206 (50.98%)	20,562 (51.88%)
1~3	2900 (7.32%)	3020 (7.62%)	3159 (7.97%)	2875 (7.25%)	2737 (6.91%)	2926 (7.38%)	2938 (7.41%)	2922 (7.37%)	2973 (7.50%)
3~15	7493 (18.90%)	7552 (19.05%)	7734 (19.51%)	7086 (17.88%)	7187 (18.13%)	7329 (18.49%)	7196 (18.16%)	7317 (18.46%)	7256 (18.31%)
15~60	6395 (16.13%)	6134 (15.48%)	5858 (14.78%)	6324 (15.96%)	6352 (16.03%)	6386 (16.11%)	6153 (15.52%)	6389 (16.12%)	6070 (15.31%)
>60	2745 (6.93%)	2699 (6.81%)	2684 (6.77%)	2725 (6.88%)	2734 (6.90%)	2756 (6.95%)	2817 (7.11%)	2802 (7.07%)	2775 (7.00%)
TOTAL	39,636 (100%)	39,636 (100%)	39,636 (100%)	39,636 (100%)	39,636 (100%)	39,636 (100%)	39,636 (100%)	39,636 (100%)	39,636 (100%)

*FPKM, Fragments per Kilobase of transcript sequence per Millions of base pairs. Ratios of gene numbers to the total gene number are presented in parentheses*.

*Interval 1–3, low expression; 3–15, medium expression; 15–60, high expression; >60, very high expression*.

### The identification of differentially expressed genes between the infected and non-infected *Cf-12* tomato

**Figure 2:**
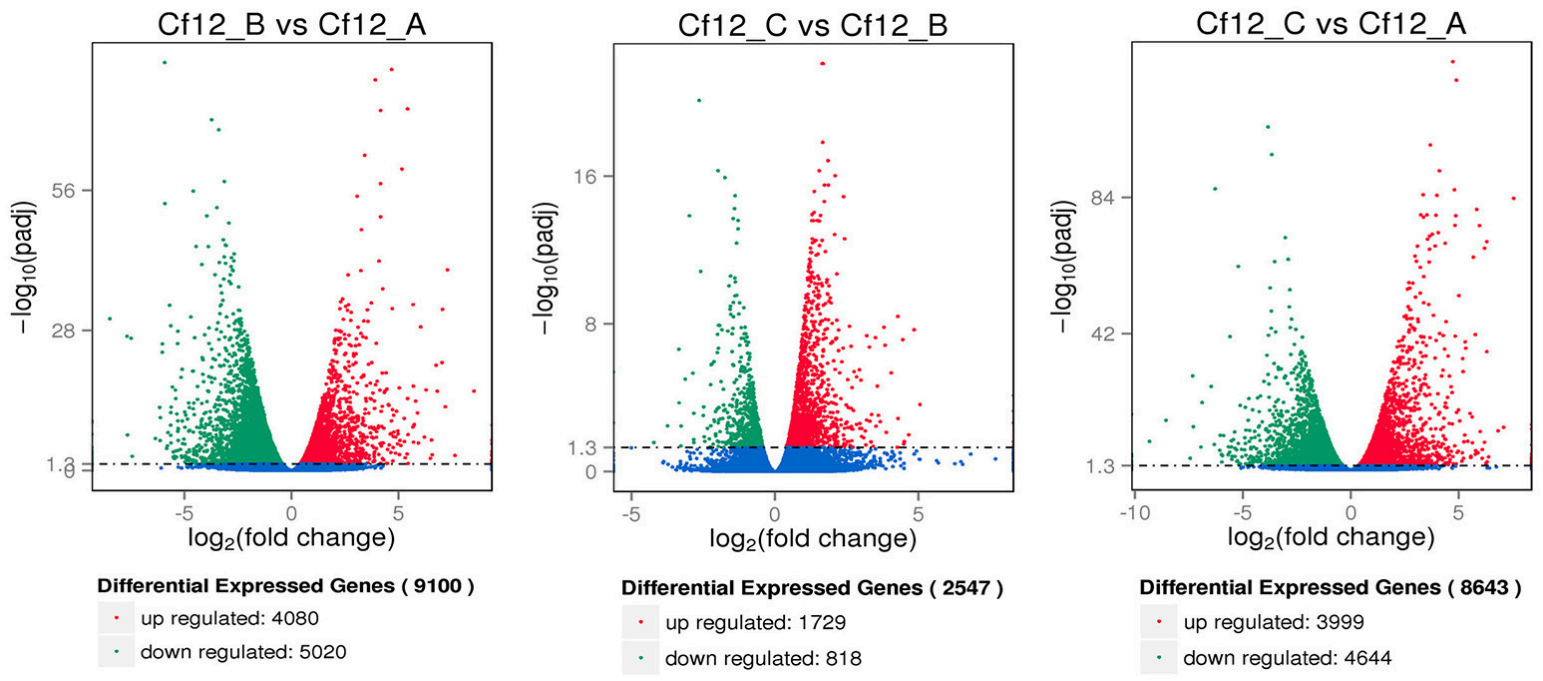
**Volcano plot showing differentially expressed genes between different libraries**. *Padj* < 0.05 was used as the threshold to judge the significance of the difference in gene expression. Red plots represent upregulated genes; green plots represent downregulated genes; blue plots represent genes with no significant difference.

**Figure 3:**
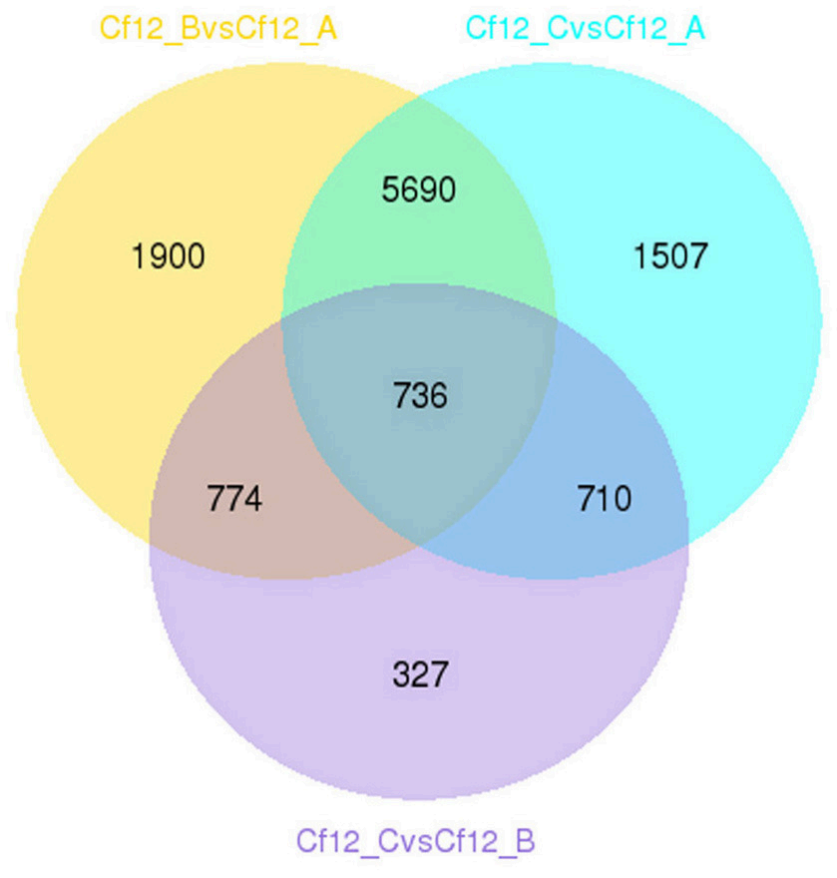
**Venn diagram of the relationship between DEG groups**. The numbers indicate the DEG number in each DEG group shown in Table S5.

**Figure 4:**
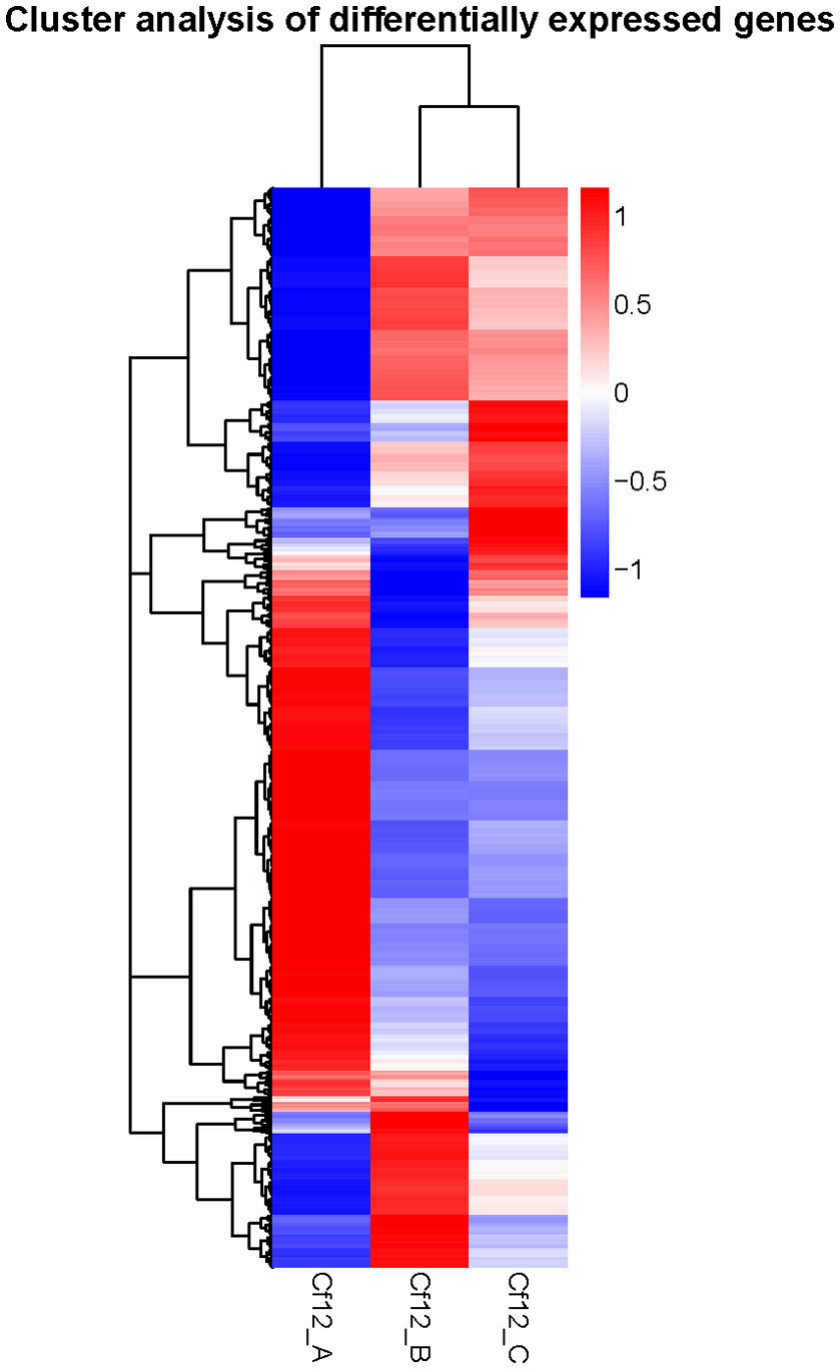
**Hierarchical clustering of DEGs**. The blue bands indicate low gene expression quantity, and the red bands represent high gene expression quantity.

DEGs were identified using the DESeq software with a *padj* <0.05. For Cf12_B vs. Cf12_A, 9100 DEGs were detected, including 4080 upregulated and 5020 downregulated DEGs (Table S5A, Figure 2); for Cf12_C vs. Cf12_A, 8643 DEGs were detected (3999 upregulated and 4644 downregulated DEGs; Table S5B, Figure 2); and for Cf12_C vs. Cf12_B, 2547 DEGs were identified (1729 upregulated and 818 downregulated DEGs; Table S5C, Figure 2). In addition, 736 DEGs were shared among the three groups (Cf12_B vs. Cf12_A, Cf12_C vs. Cf12_A, and Cf12_C vs. Cf12_B). Approximately 1500 DEGs were shared between Cf12_C vs. Cf12_A and Cf12_C vs. Cf12_B, or Cf12_B vs. Cf12_A and Cf12_C vs. Cf12_B. However, 6426 DEGs were shared between Cf12_B vs. Cf12_A and Cf12_C vs. Cf12_A. This further suggests that a common group of genes were activated or deactivated upon *C. fulvum* infection (Figure 3). In order to observe the overall changes of the quantity of gene expression, a hierarchical clustering of DEGs using FPKM analysis was performed. Results revealed that after the inoculation of *C. fulvum* on *Cf-12* tomato, genes with low expression quantity in Cf12_A increased in expression (in both Cf12_B and Cf12_C). On the contrary, many genes with high expression quantity in Cf12_A decreased in expression in Cf12_B or Cf12_C (Figure 4). Changes in gene expression quantity were also observed between Cf12_B and Cf12_C (Figure 4), suggesting that the response of *Cf-12* tomato to *C. fulvum* infection changes as time progresses.

### The identification of gene expression patterns of DEGs

**Figure 5:**
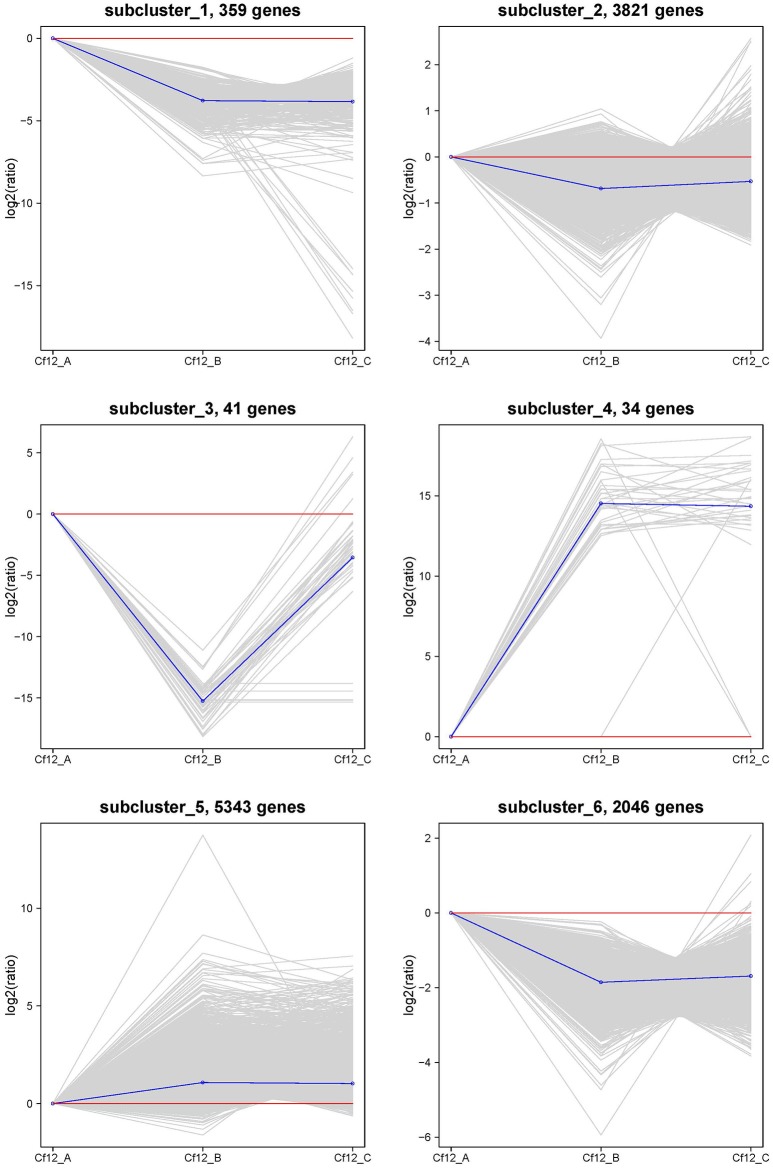
**The clustering of DEGs expression patterns**. The six expression patterns of DEGs obtained by K-means clustering algorithm is shown, which are represented as upregulated (subcluster_4 and subcluster_5), transient (subcluster_3), and downregulated (subcluster_1, subcluster_2, and subcluster_6). Expression ratios are expressed as Log_2_.

In order to identify these similar expression patterns, the relative expression level of DEGs were analyzed by K-means clustering algorithm (Hartigan and Wong, 2013). Clustering analysis revealed that six expression patterns (subclusters) of DEGs were identified (Figure 5). The most prominent group was subcluster_5, in which 5343 genes were upregulated after *C. fulvum* infection. A similar pattern was observed in subcluster_4, where genes revealed a higher expression level in Cf12_B and Cf12_C; however, the number of genes was much lesser than that in subcluster_5. Subcluster_1, subcluster_2 and subcluster_6 revealed a similar expression pattern, in which most of the genes were downregulated in Cf12_B and Cf12_C; but the downregulated level was relatively small. Subcluster_3 contained 41 genes that were downregulated in Cf12_B, but were upregulated in Cf12_C. These dynamic gene expression patterns further suggest that *Cf-12* tomato was resistant to *C. fulvum via* a highly complex process.

### Validation of RNA-Seq data by qRT-PCR

**Figure 6:**
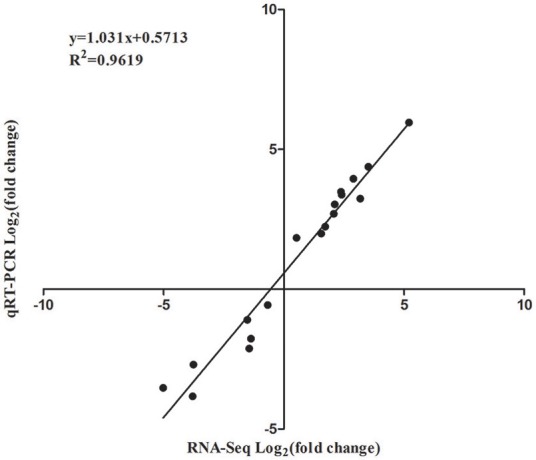
**Correlation of expression levels between RNA-Seq and qRT-PCR**.

In order to validate the RNA-Seq data, qRT-PCR was performed through 18 DEGs using three biological replicates. These 18 genes were selected to reflect some of the functional categories and pathways described below (Section GO and KEGG Enrichment Analysis of DEGs), such as the plant-pathogen interaction pathway and plant hormone signal transduction pathway (Table S1). These qRT-PCR results were compared with the RNA-Seq data. As shown in Figure 6, the trends of these gene expression patterns were consistent and had a strong positive correlation coefficient (*R*^2^ = 0.9619), indicating that the RNA-Seq data was reliable.

### GO and KEGG enrichment analysis of DEGs

In order to further characterize the functions of DEGs, GO enrichment analysis was performed using GOseq. The top 10 enrichment terms of the biological process, cellular component and molecular function were selected, respectively, as the main nodes of the directed acyclic graph. In the biological process category, significant terms were enriched in the phosphorus metabolic process, protein phosphorylation biological process, cellular protein modification process, salicylic acid metabolic process, response to jasmonic acid, and salicylic acid biosynthetic process. These processes were generally associated with disease resistance (Figure S5A). Components associated with membranes such as thylakoid, plastid thylakoid membranes, and photosynthetic membranes (Figure S5B) were significantly enriched in the cellular component category. In the molecular function category, transferase activity, phosphotransferase activity and protein serine/threonine kinase activity were enriched in catalytic activity; and purine nucleoside binding, anion binding, nucleoside binding, adenyl nucleotide binding and ATP binding were enriched in binding (Figure S5C). These binding terms and catalytic activity terms played an important role in signal recognition and signal transduction.

One interesting finding is that many transcription factor genes were significantly upregulated or downregulated in response to *C. fulvum*. A total of 406 transcription factor-related DEGs were identified and annotated into 60 families (Pérez-Rodríguez et al., 2010; Jin et al., 2014; Wang et al., 2016b; Table S6). These 60 transcription factor families were grouped into two clusters (Figure S6). The majority of DEGs in cluster I revealed an upregulation trend at the early stage (Cf12_B) and a downregulated trend at the later stages (Cf12_C). In cluster II, DEGs exhibited a higher expression at both stages or only at the later stage. For example, one of the MYB family transcription factors (Solyc05g053150.1) was upregulated to more than 5-fold in both Cf12_B vs. Cf12_A and Cf12_C vs. Cf12_A (Table S6). This protein, like most of the 406 transcription factors, remains uncharacterized; and is predicted to be a MYB23-like transcription factor. The downregulation of transcription factors was also identified. For example, the OFP family Solyc09g065350.1 (Wang et al., 2005), another uncharacterized transcription factor, was downregulated to ~5-fold in both infection stages. This result suggests that transcription factors play a critical role in response, especially in the early response, to *C. fulvum* infection.

**Figure 7:**
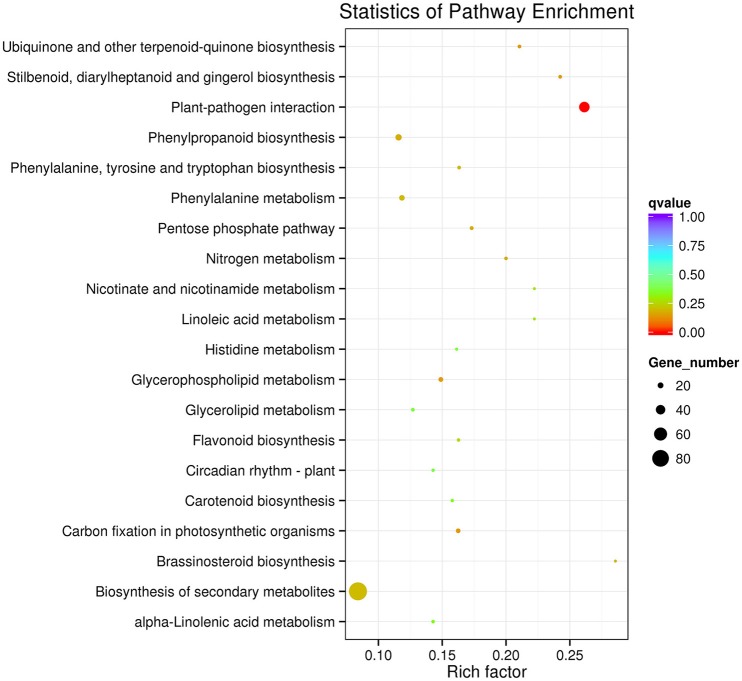
**Scatter plot of the KEGG pathway enrichment of DEGs**. Rich factor is the ratio of the DEG number to the background number in a certain pathway. The size of the dots represents the number of genes, and the color of the dots represents the range of the q-value.

The KEGG pathway enrichment analysis was used to identify the biological pathways of incompatible interaction. It was found that the plant-pathogen interaction pathway was significantly enriched (FDR ≤ 0.05). As shown in Figure 7, the number of genes and the rich factor are significantly higher than the other pathways. Many other disease-resistance pathways, including stilbenoid and gingerol biosynthesis, phenylpropanoid biosynthesis, ubiquinone and quinone biosynthesis, flavonoid biosynthesis, pentose phosphate pathway, and nitrogen metabolism were also enriched. In addition, it was found that the transduction plant hormone signals, the biosynthesis of unsaturated fatty acids, fatty acid metabolism, and carbon metabolism participate in *Cf-12* tomato response to *C. fulvum* infection.

## Discussion

In this study, we investigated the transcriptome profiles of *Cf-12* tomato in response to *C. fulvum* infection using RNA-Seq. More than 20,000 transcripts were identified from these three samples (Cf12_A, 0 dpi; Cf12_B, 4 dpi; Cf12_C, 8 dpi), in which 9100 DEGs between Cf12_B and Cf12_A, and 8643 DEGs between Cf12_C and Cf12_A were identified. GO and KEGG analyses revealed that many DEGs and their associated pathways are involved in disease resistance against fungal pathogens such as calcium dependent protein kinases (*CDPK*), wound salicylic acid inducible protein kinase (*SIPK*), respiratory burst oxidase homolog protein D/B (*Rboh*), heat shock protein 90 (*Hsp90*) and suppressor of G2 allele of SKP1 (*SGT1*; Piedras et al., 1998; Rivas and Thomas, 2005; Nekrasov et al., 2006; Hong et al., 2007).

In order to cope with the infection of *C. fulvum*, tomato plants have established a series of defense mechanisms through a complex signal transduction network. The first layer of defense is to recognize *C. fulvum* by pattern recognition receptors, and initiate the resistance response (Miya et al., 2007). In our study, chitin elicitor receptor kinase 1 (*CERK1*, Solyc07g049190.2, and Solyc07g049180.2), a pattern recognition protein, was abundantly expressed after infection. It would be interesting to conduct further studies to see whether a higher expression of CERK1 is involved in the activation of chitin signaling.

After the recognition of infection, *Cf-12* tomato quickly established complex signal defense pathways such as CDPK. In our study, CDPK (Solyc03g113390.2, Solyc10g074570.1, Solyc02g083850.2, and Solyc10g076900.1) and MEKK1 (Solyc01g104530.2, and Solyc07g053170.2) were expressed higher at the early stage of infection, and subsequently stimulated the respiratory burst oxidase homolog (Rboh, Solyc01g099620.2, and Solyc03g117980.2) at a later stage. This is consistent with previous studies suggesting that these genes play critical roles in *Cf-12* tomato response to *C. fulvum* infection.

The jasmonic acid, brassinosteroid, and ethylene pathways play important roles in the resistance to biotrophic pathogens such as downy mildew and powdery (Ellis and Turner, 2001; Walters et al., 2002). In the present study, we found that the jasmonate-zim-domain gene (*JAZ*, Solyc12g009220.1), which encodes a major protein in the jasmonic acid signaling pathway, was upregulated after *C. fulvum* infection. Brassinosteroid insensitive 1-associated receptor kinase 1 (*BAK1*, Solyc01g104970.2), brassinosteroid-signaling kinase (*BSK*, Solyc10g085000.1), ethylene receptor (*ETR*, Solyc06g053710.2) and ethylene response factor 1/2 (*ERF1/2*, Solyc09g066360.1) were also upregulated; suggesting that similar to previous studies, jasmonic acid, brassinosteroid, and ethylene may play a role in the resistance of *Cf-12* tomato to *C. fulvum*.

Physiological races of *C. fulvum* that are virulent to *Cf-2, Cf-4, Cf-5*, and *Cf-9* have been reported (Piedras et al., 1998; Romeis et al., 2001; Nekrasov et al., 2006; Hong et al., 2007; Varshney et al., 2009). However, no physiological race virulent to *Cf-12* has been identified. In the present study, we revealed that genes encoding the NPR1-like protein (Solyc07g040690.2 and Solyc02g069310.2) and the transcription factor TGA (Solyc11g068370.1 and Solyc06g074320.2) were upregulated, and both of which are involved in the salicylic acid signaling pathway. This could lead to a higher expression of pathogenesis-related protein 1 (PR1) during the disease resistance process. A previous report suggested that the salicylic acid pathway is not required for *Cf-2-* or *Cf-9-*dependent resistance to *C. fulvum* (Brading et al., 2000), suggesting that the salicylic acid pathway might be a unique pathway for *Cf-12*-dependent resistance.

Responses to infection depend on different gene expression levels, which require various transcription factors. It has been reported that many transcription factors such as WRKY, NAC, MYB, and bZIP families (Singh et al., 2002; Olsen et al., 2005) actively respond to *C. fulvum* infections. Consistent with previous reports, we found that 35 transcription factors in the MYB family were differentially expressed; and one of which (Solyc05g053150.1) had the highest level of upregulation among all transcription factor DEGs. Many TF families such as TUB, TCP, SET, SBP, PHD, and Orphans have not been reported in the regulation of tomato leaf mold disease resistance, but some of these TFs have been reported in grapevine (*Vitis amurensis*) against downy mildew (Li et al., 2015) and in ramie against root-lesion nematode infection (Zhu et al., 2014). In addition, majority of the DEGs reported in this study remain uncharacterized. Further studies of these transcription factors related DEGs could greatly help understand the molecular mechanism of the defense response to *C. fulvum* infection.

In summary, based on our microscopic data and GO/KEGG analysis of RNA-seq data, we believe that the *Cf-12* tomato response to *C. fulvum* infection follows several steps. When the mycelium of *C. fulvum* grows into the interspace of the stomata and mesophyll cells, the effector proteins secreted by *C. fulvum* are rapidly recognized by *Cf-12* tomato. This triggers downstream defense signaling transductions associated with the Ca^2+^ channel, as well as several pathways, including pathways involving jasmonic acid, brassinosteroid and ethylene. Then, these defense-related transcription factors (TFs) such as MYB proteins (Moore et al., 2011; van Verk et al., 2011; Puranik et al., 2012) are triggered; which actively regulate downstream resistance pathways. Finally, hypersensitive necrosis occurs locally (Hammond-Kosack and Jones, 1996), and the *C. fulvum* hyphae is restricted only in infected areas. This study facilitates our understanding of the molecular mechanism of *Cf-12* tomato against *C. fulvum* infection.

## Database link and accessions

The raw sequencing data of the nine samples have been submitted to the NCBI Sequence Read Archive (SRA, http://www.ncbi.nlm.nih.gov/sra). The accession numbers are: SRR4041970, SRR4041973, SRR4041974, SRR4041975, SRR4042017, SRR4042029, SRR4042030, SRR4042031, and SRR4042032.

## Author contributions

DX, JL, XX, and JJ conceived and designed the experiments; HZ and XFC performed the RNA isolation and qRT-PCR experiments; DX and XLC performed the data analysis; and DX wrote the manuscript. All authors read and approved the final manuscript.

### Conflict of interest statement

The authors declare that the research was conducted in the absence of any commercial or financial relationships that could be construed as a potential conflict of interest.

## Supplementary material

Figure S1 **Classification of raw reads**.

Figure S2 **RNA-Seq correlation analysis between samples**.

Figure S3 **The percentage of reads mapped to genome regions**.

Figure S4 **Reads density on chromosomes of the tomato reference genome**.

Figure S5 **Significant enrichment of GO terms. (A)** GO significant enrichment in biological process; **(B)** GO significant enrichment in cellular component; **(C)** GO significant enrichment in molecular function.

Figure S6 **Response of transcription factors to ***C. fulvum*** infection**. Each line color corresponds to the same gene from Cf12_B/Cf12_A and Cf12_C/Cf12_A. The expression level of the same gene is represented by different colors.

Table S1 **Primers used for qPCR**.

Table S2 **Summary of RNA-Seq data and mapping**.

Table S3 **Structural optimization of known genes**.

Table S4 **Results of the new transcription structure annotation**.

Table S5 **DEGs between two samples. (A)** DEGs between Cf12_B and Cf12_A; **(B)** DEGs between Cf12_C and Cf12_A; **(C)** DEGs between Cf12_C and Cf12_B.

Table S6 **Response of transcription factors to ***C. fulvum*** infection**.
