# Supplementary material for: Transcriptome Analysis of the Cf-12-Mediated Resistance Response to Cladosporium fulvum in Tomato
Source: Front Plant Sci. 2017 Jan 5;7:2012. doi: 10.3389/fpls.2016.02012 (PMC5212946; doi:10.3389/fpls.2016.02012)
Supplement: Figure S1 — Classification of raw reads. [file Image1.PDF]

Figure S1

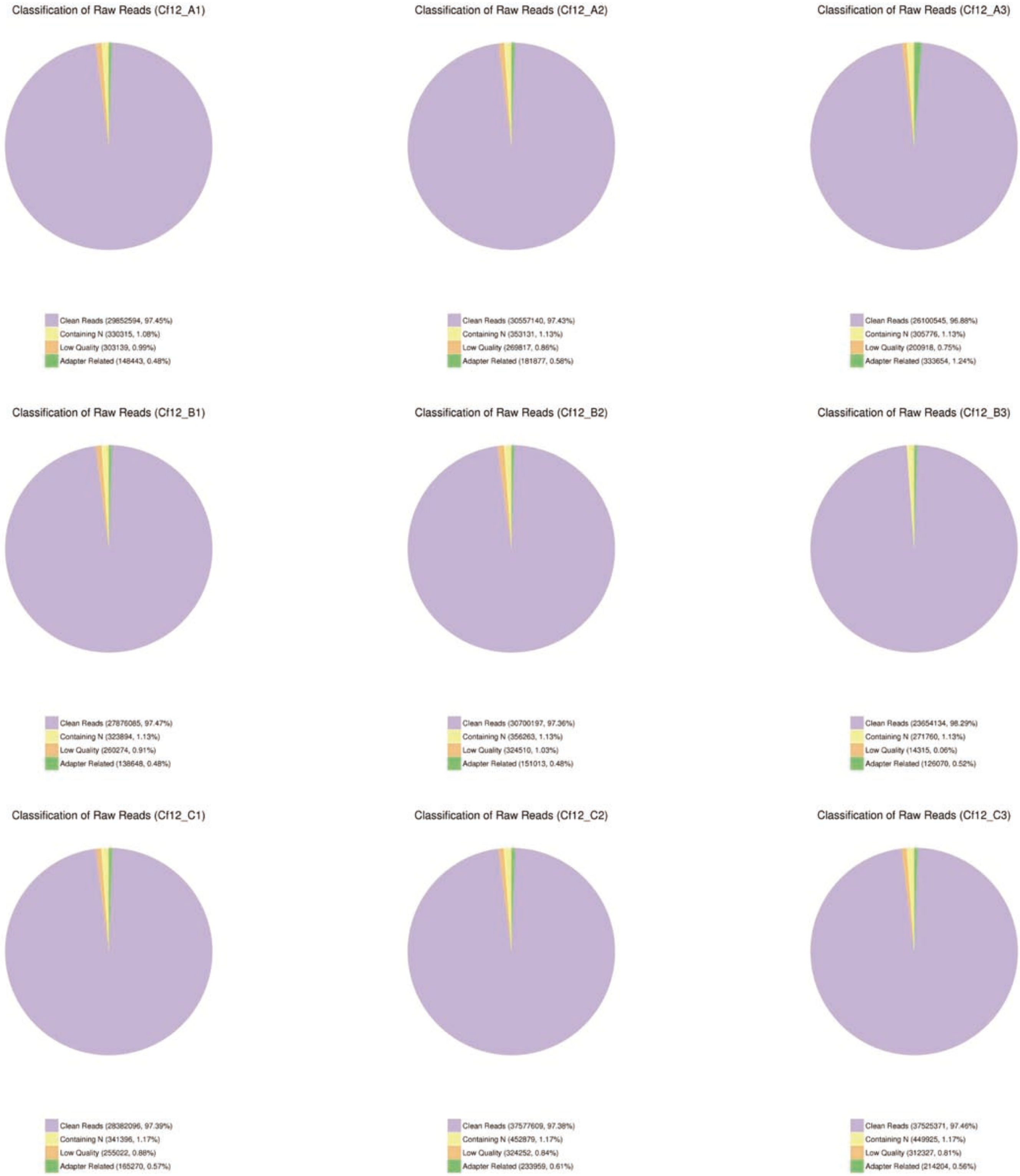

Classification of Raw Reads (Cf12_A1)
Clean Reads (29852594, 97.45%)
Containing N (330315, 1.08%)
Low Quality (303139, 0.99%)
Adapter Related (148443, 0.48%)
Classification of Raw Reads (Cf12_A2)
Clean Reads (30557140, 97.43%)
Containing N (353131, 1.13%)
Low Quality (269817, 0.86%)
Adapter Related (181877, 0.58%)
Classification of Raw Reads (Cf12_A3)
Clean Reads (26100545, 96.88%)
Containing N (305776, 1.13%)
Low Quality (200918, 0.75%)
Adapter Related (333654, 1.24%)
Classification of Raw Reads (Cf12_B1)
Clean Reads (27876085, 97.47%)
Containing N (323894, 1.13%)
Low Quality (260274, 0.91%)
Adapter Related (138648, 0.48%)
Classification of Raw Reads (Cf12_B2)
Clean Reads (30700197, 97.36%)
Containing N (356263, 1.13%)
Low Quality (324510, 1.03%)
Adapter Related (151013, 0.48%)
Classification of Raw Reads (Cf12_B3)
Clean Reads (23654134, 98.29%)
Containing N (271760, 1.13%)
Low Quality (14315, 0.06%)
Adapter Related (126070, 0.52%)
Classification of Raw Reads (Cf12_C1)
Clean Reads (28382096, 97.39%)
Containing N (341396, 1.17%)
Low Quality (255022, 0.88%)
Adapter Related (165270, 0.57%)
Classification of Raw Reads (Cf12_C2)
Clean Reads (37577609, 97.38%)
Containing N (452879, 1.17%)
Low Quality (324252, 0.84%)
Adapter Related (233959, 0.61%)
Classification of Raw Reads (Cf12_C3)
Clean Reads (37525371, 97.46%)
Containing N (449925, 1.17%)
Low Quality (312327, 0.81%)
Adapter Related (214204, 0.56%)
