# Supplementary figures and images for: Transcriptome Analysis of the Cf-12-Mediated Resistance Response to Cladosporium fulvum in Tomato
Source: Front Plant Sci. 2017 Jan 5;7:2012. doi: 10.3389/fpls.2016.02012 (PMC5212946; doi:10.3389/fpls.2016.02012)

Figure S2

## Pearson correlation between samples

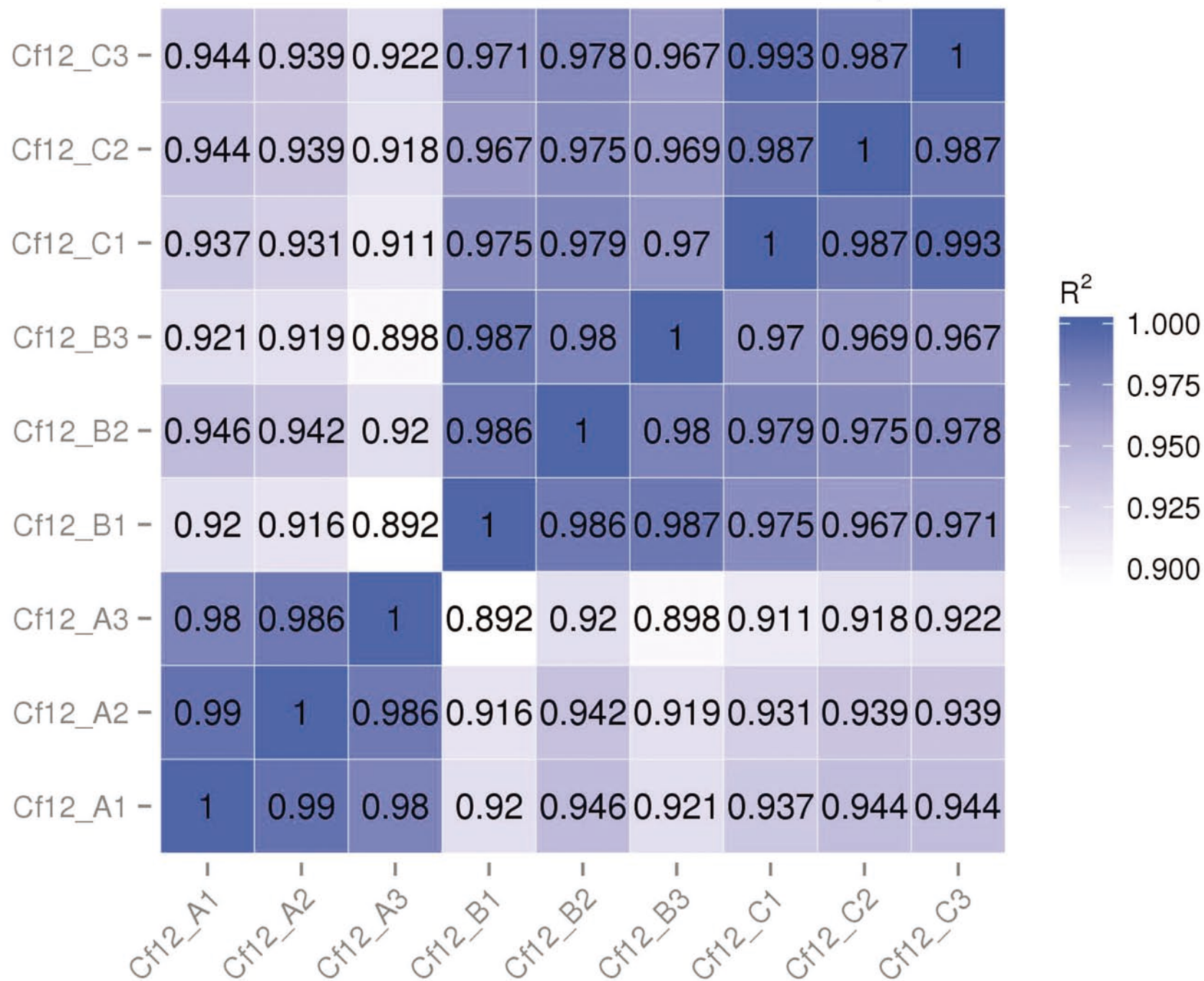

Supplement: Figure S2 — RNA-Seq correlation analysis between samples. [file Image2.PDF]

Percent of reads mapped to genome regions (Cf12\_C3)

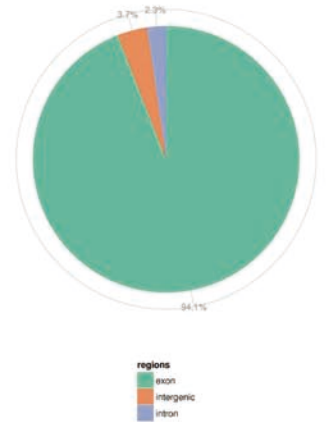

Supplement: Figure S3 — The percentage of reads mapped to genome regions. [file Image3.PDF]

Figure S5a

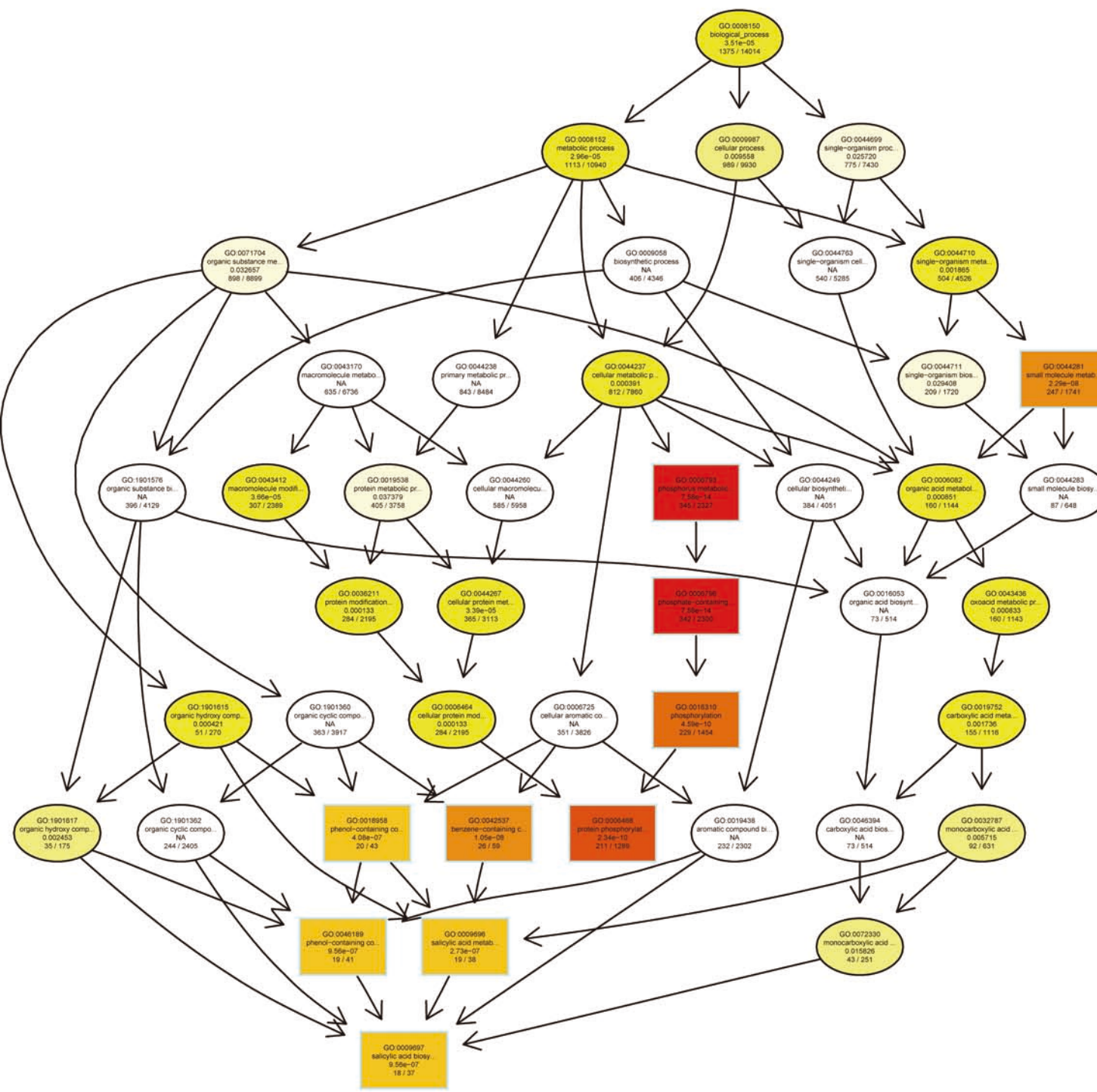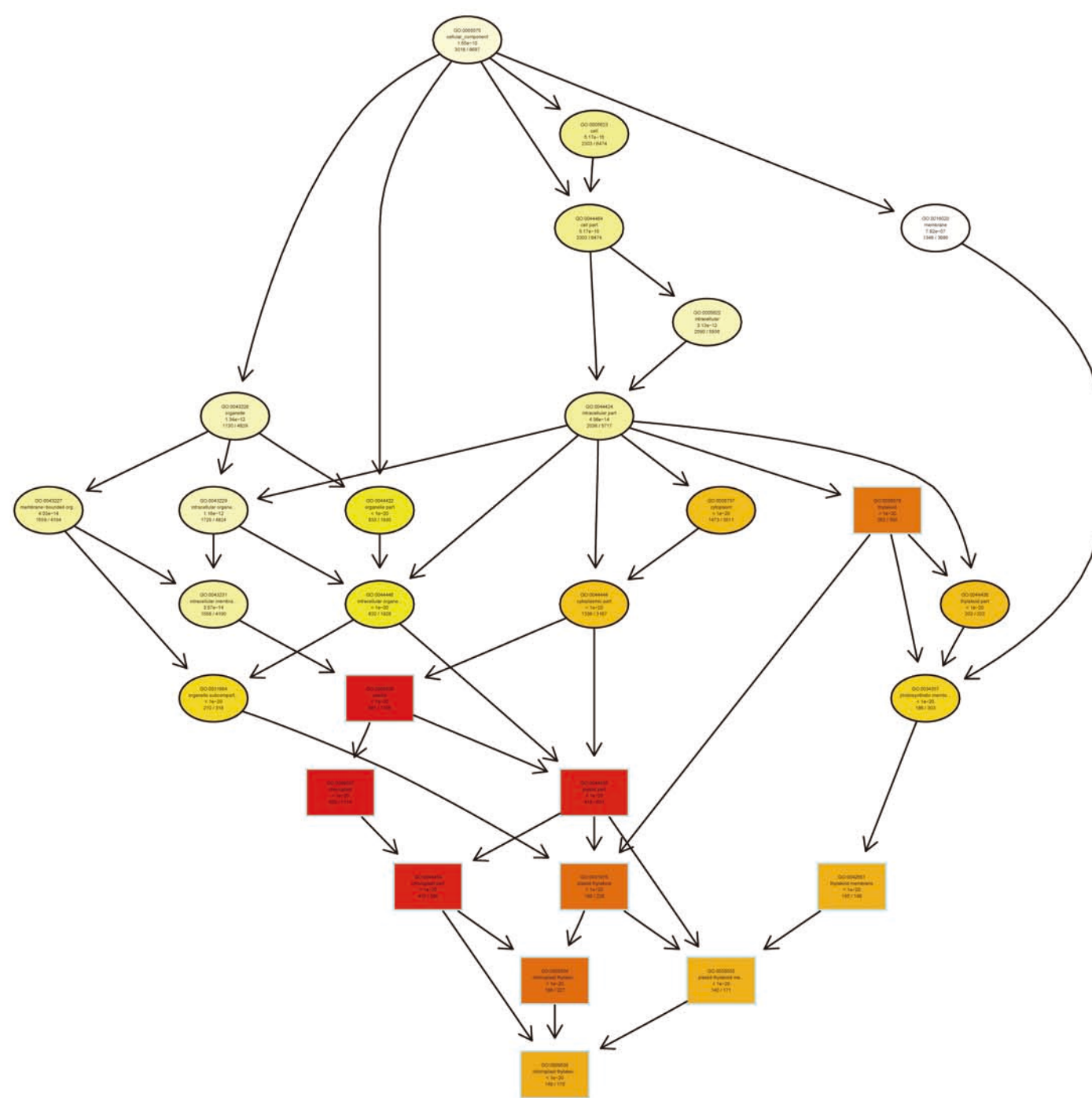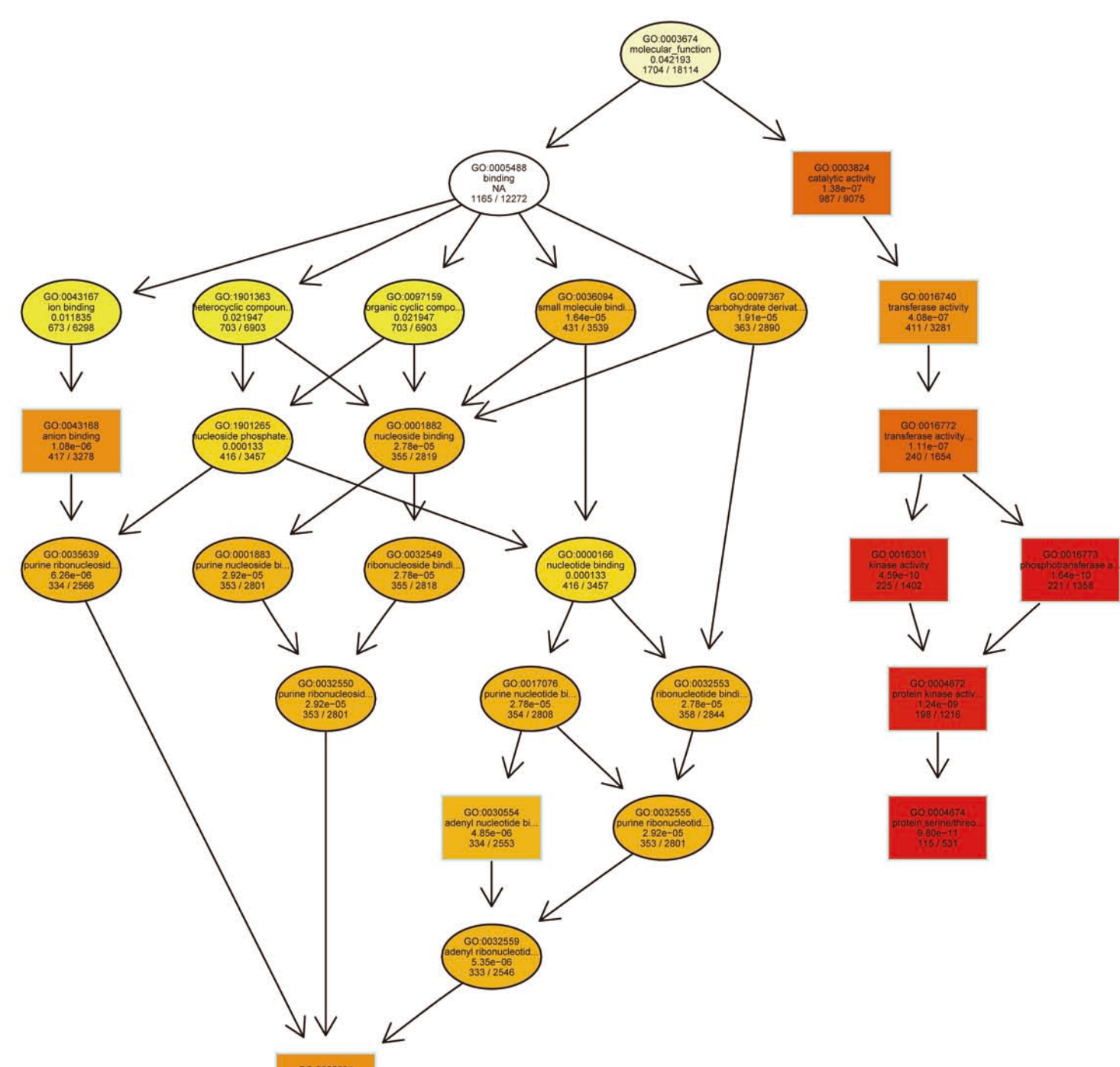

Supplement: Figure S5 — Significant enrichment of GO terms. (A) GO significant enrichment in biological process; (B) GO significant enrichment in cellular component; (C) GO significant enrichment in molecular function. [file Image5.PDF]

Figure S6

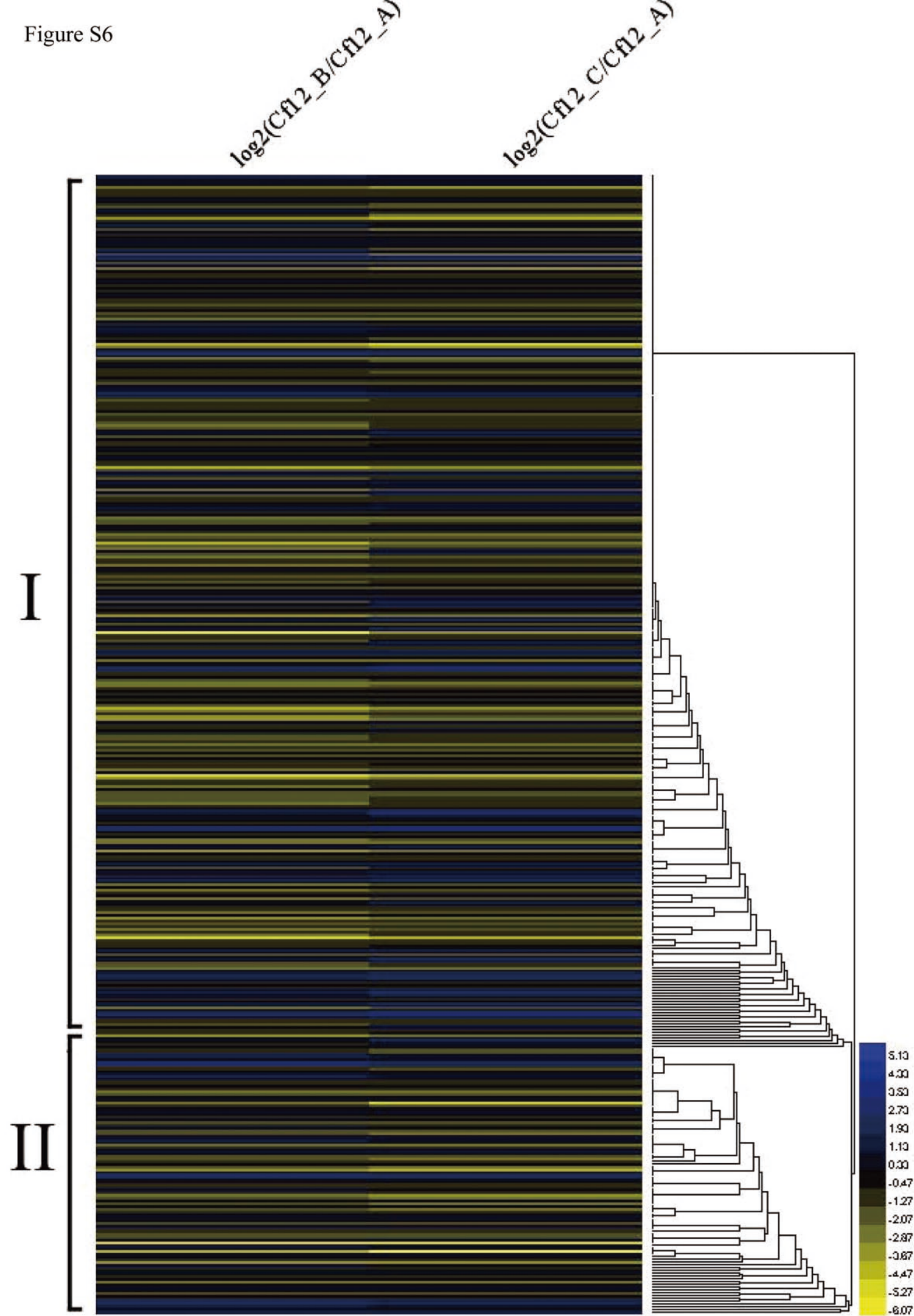

Supplement: Figure S6 — Response of transcription factors to C. fulvum infection. Each line color corresponds to the same gene from Cf12_B/Cf12_A and Cf12_C/Cf12_A. The expression level of the same gene is represented by different colors. [file Image6.PDF]
