# Supplementary material for: Transcriptome Analysis of the Cf-12-Mediated Resistance Response to Cladosporium fulvum in Tomato
Source: Front Plant Sci. 2017 Jan 5;7:2012. doi: 10.3389/fpls.2016.02012 (PMC5212946; doi:10.3389/fpls.2016.02012)
Supplement: Figure S4 — Reads density on chromosomes of the tomato reference genome. [file Image4.PDF]

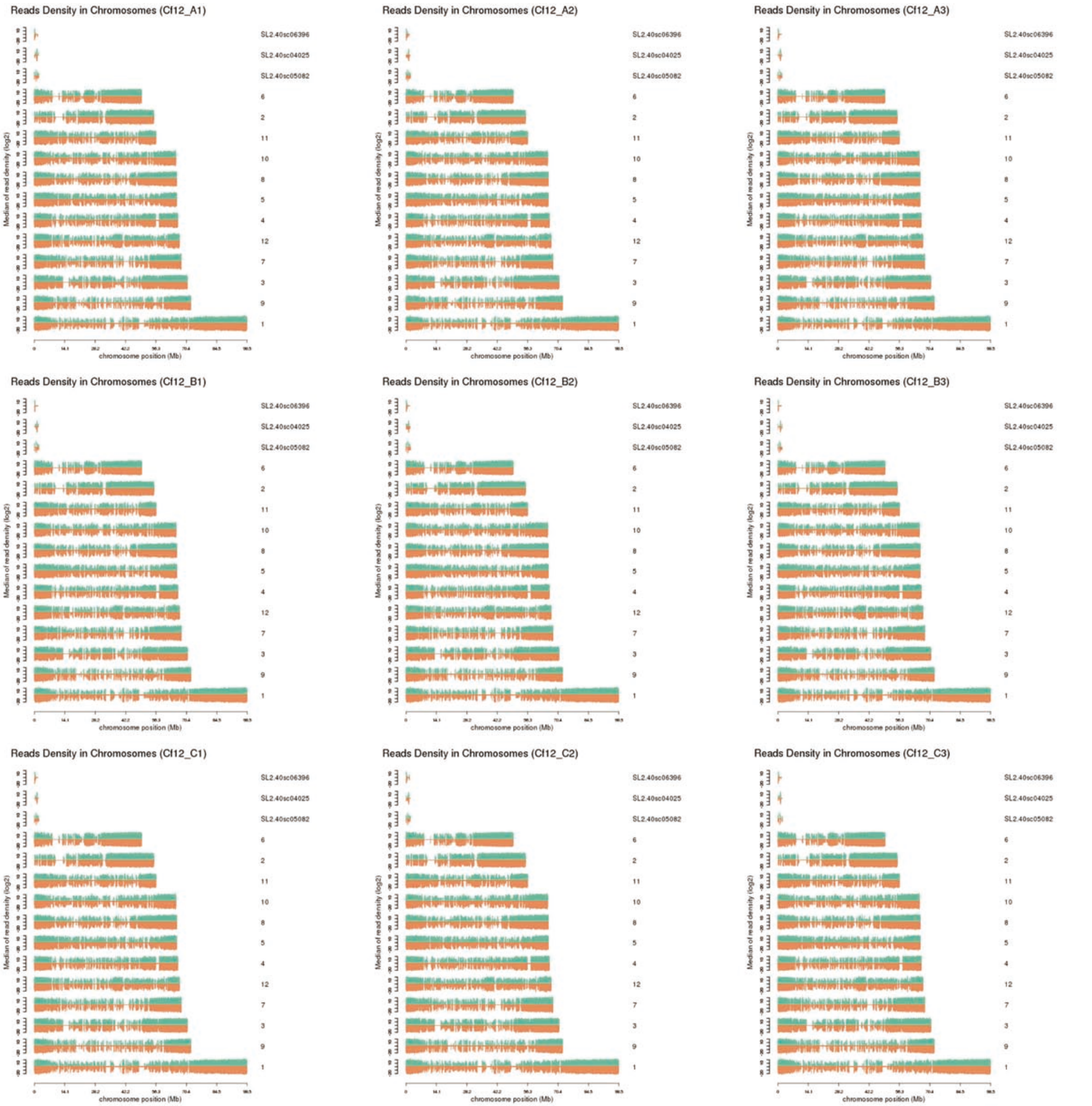

Reads Density in Chromosomes (Cf12_A1)
Reads Density in Chromosomes (Cf12_A2)
Reads Density in Chromosomes (Cf12_A3)
Reads Density in Chromosomes (Cf12_B1)
Reads Density in Chromosomes (Cf12_B2)
Reads Density in Chromosomes (Cf12_B3)
Reads Density in Chromosomes (Cf12_C1)
Reads Density in Chromosomes (Cf12_C2)
Reads Density in Chromosomes (Cf12_C3)
Median of read density (log2)
SL2.40sc06396
SL2.40sc04025
SL2.40sc05082
6
2
11
10
8
5
4
12
7
3
9
1
0
14.1
28.2
42.2
56.3
70.4
84.5
98.5
chromosome position (Mb)
