# Supplementary material for: Transcriptome Analysis of the Cf-12-Mediated Resistance Response to Cladosporium fulvum in Tomato
Source: Front Plant Sci. 2017 Jan 5;7:2012. doi: 10.3389/fpls.2016.02012 (PMC5212946; doi:10.3389/fpls.2016.02012)
Supplement: Table S1 — Primers used for qPCR. [file Table1.PDF]

Table S1. Primers used for qPCR

Gene names	Forward primer (5'-3')	Reverse primers (5'-3')	Size (bp)
actin	TTCCGTTGCCCTGAAGTCCT	AGTTGAGCCACCACTAAGCAC	150
sly:101248095	ACTATGATGGAAGTTGTGGACGC	TTCTGGTGTTGCCTTTGTGC	123
sly:101261765	GCACCCTCCAACTCCACTAAT	GTCCCTTCATGTCAACATCCTC	191
sly:101268153	ACTCCGTTTATGGACTTACCTGC	TTGGCTTGGCGTTGTCTTC	182
sly:101259966	TTCCTTTCTGATGCTGGAGTTC	GGTTGAGTCGTAGTGAGGCAGT	180
sly:101263535	AACAATGAAGCCTGTAGCAA	GCAATCACGAACGGCTCA	216
sly:100135703	AGAAATACACCGAGGCAACAGA	CCAAGAGGCTTTGAAACACCAG	231
sly:101268663	TTGCCTCTATGGAGAAGTGGTG	CCAGTGAGTTCAGTTGCGATTAC	242
sly:101251136	CCTTTATTACTGCTGCTAGGTCT	CTAAGGAAACAGTAACCCCAT	166
sly:101260143	CTTTATTCGCCTTGTTCCTGAC	GATACGCAGAGTAGAAACCCACA	200
sly:101247513	TATGTGCCTCGGTGAAATGTG	CCTTGTCCTTCTGTTCCTGGTAG	121
sly:544226	TCAGTGCTGGTTGATTTCCTTG	GGGGCAGACAGATTCATTCC	101
sly:101254402	TTCGCTCATCCCTCTACTGGT	TCACTAACTCATTCCGCTGCTC	193
sly:543588	CGTCCTTTTGGATCTTCACCT	CGATACCTGGCAATAGCACTG	191
sly:544165	GCTGGGGAGGAGAATAATAACA	CCCGAAGAAGGCAAAACTG	110
sly:101243703	CAGCTAAAGTCTCCGGCGAT	ATCTAGGGCCGTTTGATTCCAC	191
sly:101244669	ACATGAACAGTACAAATGGCAAG	ACAATGCGTCCAGTAATTGGTC	167
sly:101245163	ATGTAGACGCAAACTCATCGC	GCATCTTCAGGGGACAATCCA	147
sly:101263402	GCAGCAACAAAAGGAGCAAT	GGCGGTATCAATAATCCAAGGT	108

Note: The GenBank accession number of *actin* is U60478.1, and was taken for internal control. The accession numbers beginning with *sly* are from KEGG Genes (http://www.genome.jp/). The relative expression levels were evaluated by $2^{-\Delta\Delta Ct}$ method.
